# Supplementary figures and images for: Transcriptome profiling helps to elucidate the mechanisms of ripening and epidermal senescence in passion fruit (Passiflora edulia Sims)
Source: PLoS One. 2020 Sep 25;15(9):e0236535. doi: 10.1371/journal.pone.0236535 (PMC7518611; doi:10.1371/journal.pone.0236535)

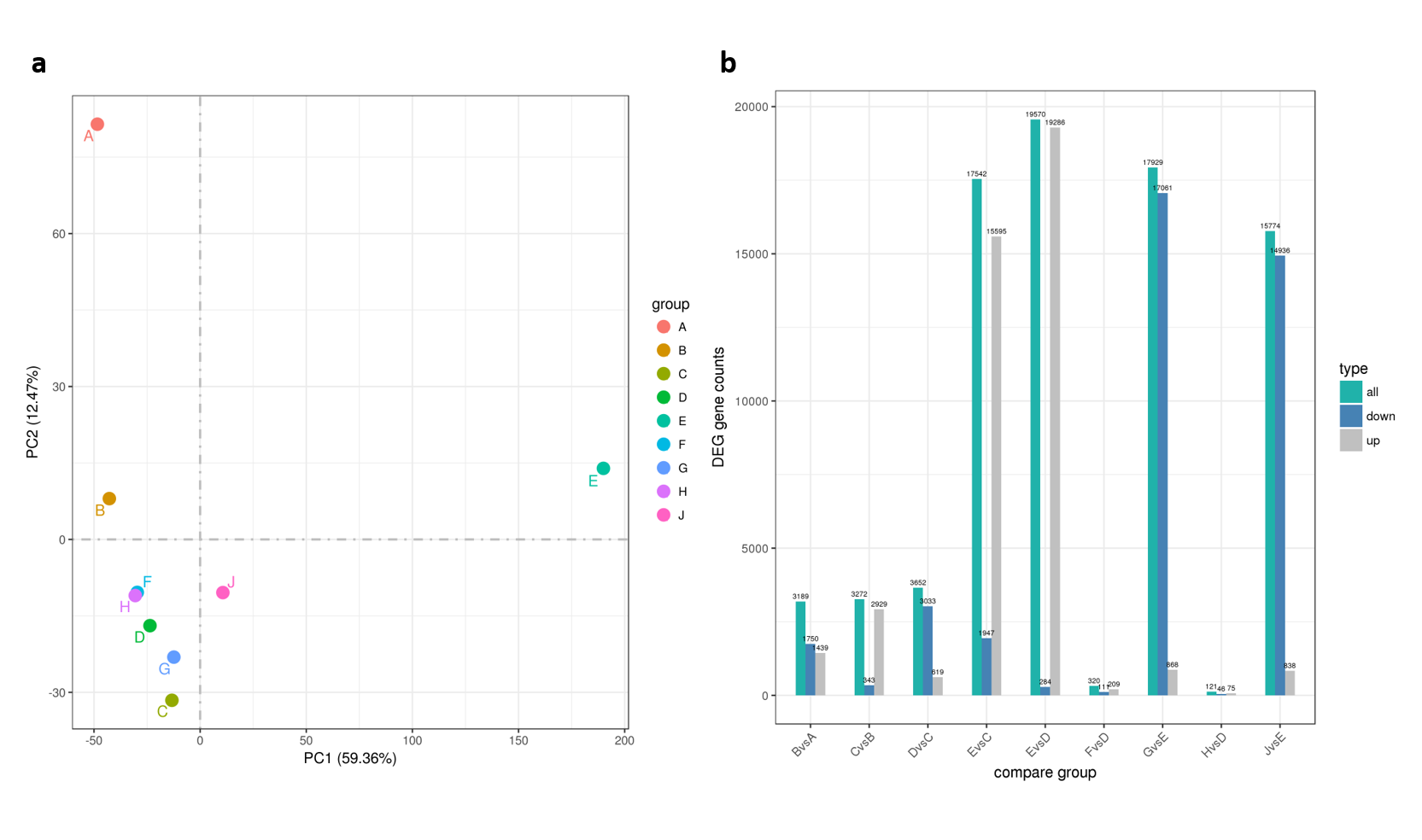

Supplement: S1 Fig — (a) Principal component analysis of the RNA-seq profile at different developmental stages of passion fruit, and postharvest stages with different treatment. (b) The DEGs counts of different comparison groups, grey indicates up-regulated and blue represents down-regulated. (TIF) [file pone.0236535.s001.tif]

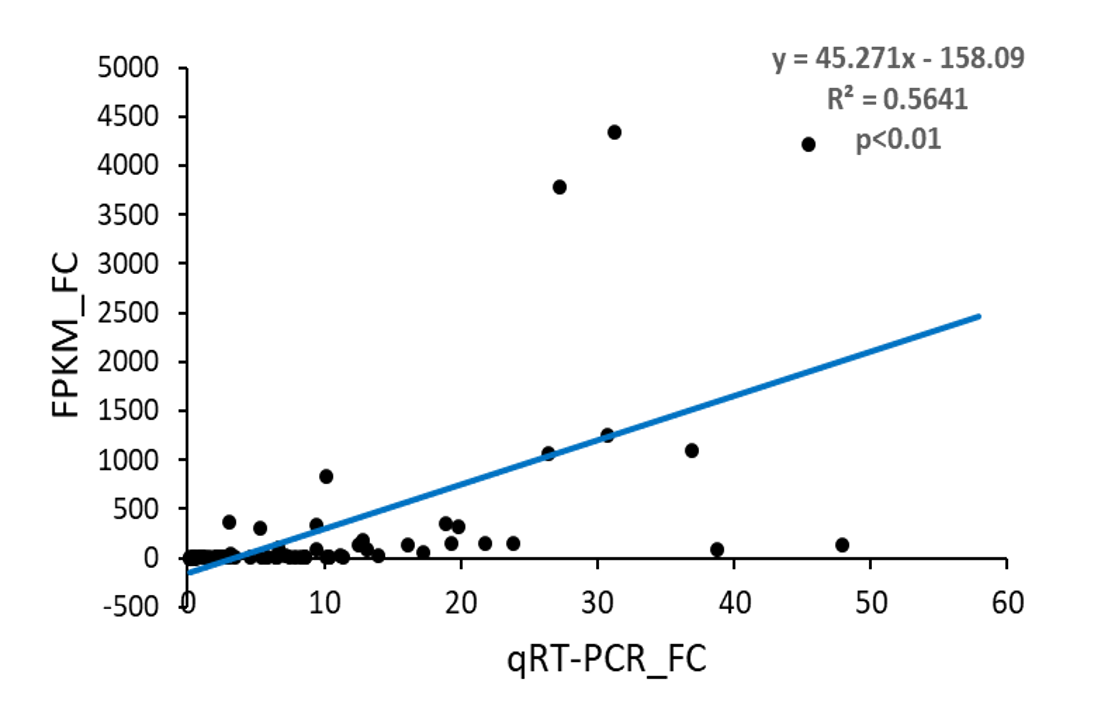

Supplement: S2 Fig — Each point represents a fold change value of expression level between the corresponding FPKM and RT-PCR in passion fruit. (TIF) [file pone.0236535.s002.tif]
