# Supplementary material for: Transcriptome profiling helps to elucidate the mechanisms of ripening and epidermal senescence in passion fruit (Passiflora edulia Sims)
Source: PLoS One. 2020 Sep 25;15(9):e0236535. doi: 10.1371/journal.pone.0236535 (PMC7518611; doi:10.1371/journal.pone.0236535)
Supplement: Attachment — Submitted filename: Response Reviewer #2.docx [file pone.0236535.s005.docx]

**Dear reviewer,**

Thank you very much for your patience to read our article and putting forward certain key suggestions for modification. We have made explanations for your questions and revised the insufficient parts of the article according to your suggestions. The following is our reply to your questions one by one.

**1: What is novel in this study? What is explained in the manuscript is already well known in several other studies with different fruits.**

**Response:** Thank you very much for your very professional questions. The novel in this article is the first systematic comparison and analysis of different storage periods of passion fruit from immature to mature period and 1-MCP and preservative film (PF) treatment after picking. Although 1-MCP treatments have been extensively studied in other fruits, they have rarely been compared with PF treatment, and our results show that PF treatment appears to be more cost-effective than 1-MCP treatment (further research is needed to verify this conclusion). At present, a growing number of people advocate green healthy food that without food chemical additives and chemical pesticides. This is one of the reasons why we will carry out further research. Thanks again for your patience. If you have any question, please contact us. We will deal with it seriously and revise it seriously.

**2: The expression of genes in KEGG classification (Fig 3B) does not match with Fig 4B. Explain.**

**Response:** Thank you very much for your attention and asking the key questions. First of all, we apologize for the careless of not clearly describing and explaining figure 3B and 4B in the manuscript. Figure 3B shows the KEGG classification of all genes detected by the transcriptome (these genes include differentially expressed genes and non-differentially expressed genes). Fig 4B shows the top 20 KEGG enrichment of differentially expressed genes in passion fruit during ripening stage. The KEGG classification in Fig 3B belongs to a large category, and each category contains several KEGG pathways of minor classification, namely the specific pathway shown in Fig 4B. Figure 3B contains Figure 4B, so it is possible that the result of figure 4B does not exactly same as Figure 3B. It is our mistake that makes you unable to understand this paper. Therefore, we have revised this manuscript and the legend of picture. Thanks again for your patience. If you have any question, please contact us. We will deal with it seriously and revise it seriously.

**3: Legend of Figure 5 is confusing. A, B and C is confusing between A, B and C of each panel. The given pathway is also not been mentioned in the legend**

**Response:** Thank you very much for your professional suggestions. To prevent confusion among readers, we changed the ‘ABCD’ in the figure to ‘abcd’ to distinguish the sample name from the figure and manuscript. The flavonoids and anthocyanin biosynthesis, carotenoids biosynthesis and phenylpropanoid biosynthesis pathway in Fig 5 were all from the results of Fig 4. The flavonoids and anthocyanin biosynthesis pathway were the downstream of phenylpropanoid biosynthesis pathway. I'm sorry that we failed to explain the logical relationship clearly, which caused you a misdirection. The legend of Fig 5 was also revised according to your suggestion. Thanks again for your patience. If you have any question, please contact us. We will deal with it seriously and revise it seriously.

**4: When the hormone response was not observed in KEGG classification (Fig 3B), then why it is studied in Fig 7B?**

**Response:** Thank you very much for your professional suggestions. Figure 3B shows the KEGG classification of all expressed genes (include differentially expressed genes and non-differentially expressed genes) detected by RNA-Seq. The signal transduction in Fig 3B is a large classification that containing the plant hormone signal transduction in Fig 7B. I'm sorry that our carelessness caused you to have doubts about the logic of the article. We have revised the manuscript according to your question. We hope you can be satisfied with the revised version this time. Thanks again for your patience. If you have any question, please contact us. We will deal with it seriously and revise it seriously.

**5: The manuscript lacks in correlation between different components, which can be improved in the revised one.**

**Response:** Thank you very much for your professional suggestions. We are very sorry for your misunderstanding of this article due to our carelessness. We have tried our best to revise this article to make the logic of the manuscript more meticulous and the language more coherent according to your advice. We sincerely hope that you will be satisfied with the revised version. Thanks again for your patience. If you have any question, please contact us. We will deal with it seriously and revise it seriously.
